# Predictors of Mortality in Acute Myocardial Infarction Complicated by Cardiogenic Shock despite Intra-Aortic Balloon Pump: Opportunities for Advanced Mechanical Circulatory Support in Asia

**DOI:** 10.3390/life14050577

**Published:** 2024-04-30

**Authors:** Weiqin Lin, Alfred Chung Lum Yip, Robin Cherian, Siew Pang Chan, Lauren Kay Mance Evangelista, Novi Yanti Sari, Hwei Sung Ling, Yoke Ching Lim, Raymond Ching Chiew Wong, Benjamin Wei Liang Tung, Li-Ling Tan, Adrian F. Low, Anand Adinath Ambhore, Shir Lynn Lim

**Affiliations:** 1 Department of Cardiology, National University Heart Centre, Singapore 119074, Singaporerobin_cherian@nuhs.edu.sg (R.C.); lmevangelista@dlsmhsi.edu.ph (L.K.M.E.); hsling@unimas.my (H.S.L.); adrian_low@nuhs.edu.sg (A.F.L.);; 2 Yong Loo Lin School of Medicine, National University of Singapore, Singapore 117597, Singapore; mdccsp@nus.edu.sg; 3 Cardiovascular Research Institute, National University Heart Centre, Singapore 119074, Singapore; 4 De La Salle Medical and Health Sciences Institute, Dasmarinas 4114, Philippines; 5 Department of Cardiology, University of the Philippines—Philippine General Hospital, Manilla 1000, Philippines; 6 Dr Mohammad Hoesin General Hospital Palembang, South Sumatra, Kota Palembang 30126, Indonesia; 7 Department of Medicine, Faculty of Medicine and Health Science, Universiti Malaysia Sarawak, Kota Samarahan 94300, Malaysia; 8 Department of Cardiology, Sarawak Heart Centre, Kota Samarahan 94300, Malaysia

**Keywords:** acute myocardial infarction, cardiogenic shock, catheter-based left ventricular assist device, mechanical circulatory support

## Abstract

**Introduction:** Acute myocardial infarction complicated by cardiogenic shock (AMI-CS) mortality remains high despite revascularization and the use of the intra-aortic balloon pump (IABP). Advanced mechanical circulatory support (MCS) devices, such as catheter-based ventricular assist devices (cVAD), may impact mortality. We aim to identify predictors of mortality in AMI-CS implanted with IABP and the proportion eligible for advanced MCS in an Asian population. **Methods:** We retrospectively analyzed a cohort of Society for Cardiovascular Angiography and Intervention (SCAI) stage C and above AMI-CS patients with IABP implanted from 2017–2019. We excluded patients who had IABP implanted for indications other than AMI-CS. Primary outcome was 30-day mortality. Binary logistic regression was used to calculate adjusted odds ratios (aOR) for patient characteristics. **Results:** Over the 3-year period, 242 patients (mean age 64.1 ± 12.4 years, 88% males) with AMI-CS had IABP implanted. 30-day mortality was 55%. On univariate analysis, cardiac arrest (*p* < 0.001), inotrope/vasopressor use prior to IABP (*p* = 0.004) was more common in non-survivors. Non-survivors were less likely to be smokers (*p* = 0.001), had lower ejection fraction, higher creatinine/ lactate and lower pH (all *p* < 0.001). On multi-variate analysis, predictors of mortality were cardiac arrest prior to IABP (aOR 4.00, CI 2.28–7.03), inotrope/vasopressor prior to IABP (aOR 2.41, CI 1.18–4.96), lower arterial pH (aOR 0.02, CI 0.00–0.31), higher lactate (aOR 2.42, CI 1.00–1.19), and lower hemoglobin (aOR 0.83, CI 0.71–0.98). Using institutional MCS criteria, 106 patients (44%) would have qualified for advanced MCS. **Conclusions:** Early mortality in AMI-CS remains high despite IABP. Many patients would have qualified for higher degrees of MCS.

## 1. Introduction

Cardiogenic shock (CS) represents the clinical expression of circulatory failure, consequent to left, right, or biventricular dysfunction. Acute myocardial infarction (AMI) is the most common etiology, accounting for 81% of patients in CS [1]. Aside from early revascularization, no other intervention has been shown to conclusively improve mortality outcomes in AMI-CS, which has remained dismal with no appreciable improvement over the past decade [2,3,4]. Patients presenting with CS in more recent times have also been observed to be more complicated, having more premorbid conditions [5]. As such, there is a definite need for more sophisticated methods of supporting the circulatory system of our current day CS patients.

Inotropes and vasopressors are commonly used in the management of AMI-CS. However, medical therapy alone may not offer sufficient circulatory support. In addition, some commonly used medications, such as dopamine and dobutamine, increase myocardial oxygen consumption and arrhythmia risks [6]. Mechanical circulatory support (MCS) devices, when instituted in a timely manner and in the right patient population, may be beneficial in the management of AMI-CS [7]. IABP utilizes a counter-pulsation technique to increase coronary artery perfusion, reduce myocardial oxygen consumption, reduce afterload and augment cardiac output (0.5–1.0 L/min) [8]. However, its use in CS has not been supported by randomized trials, resulting in a downgrading of the IABP recommendation in international guidelines; the European Society of Cardiology ST-elevation myocardial infarction (STEMI) management guidelines 2017 gave routine IABP use in CS a class III recommendation [9].

Newer devices are now available for use in managing AMI-CS. The Impella^®^ (Abiomed, Danvers, MA, USA) family of catheter-based left ventricular assist devices (cVADs) are now being used with increasing frequency around the world, including Asia [8,10,11]. They provide greater hemodynamic support compared to the IABP and can be inserted quickly at the time of coronary angiogram. Subsequent care is also simpler compared to ECMO. Observational data from the Detroit Cardiogenic Shock Initiative and the subsequent National Cardiogenic Shock Initiative have been encouraging, with reported patient survival of 76% and 72%, respectively [12,13]. Both initiatives advocate careful patient selection for cVAD use, strict adherence to a protocol and multi-disciplinary management of AMI-CS. This is a marked improvement from historical survival rates for AMI-CS. However, cost and complication risks have led to limited use of the cVAD, especially in Asia [14]. It becomes important, therefore, to identify the proportion of patients who survive with current management and identify those who may benefit from higher levels of support, as more countries in Asia start to adopt newer strategies of MCS.

In this study, we aim to identify rates and predictors of mortality in a contemporary cohort of patients with Society for Cardiovascular Angiography and Intervention (SCAI) stage C and above AMI-CS at an Asian center, prior to introduction of cVADs [15]. We will also evaluate the proportion of patients in this cohort who met selection criteria set out by the above two initiatives, potentially allowing us to understand the percentage of patients in our AMI-CS cohort who might have derived benefits from protocolized cVAD use.

## 2. Methods

### 2.1. Study Design and Population

This is a retrospective study of a cohort of multi-ethnic Asian patients with AMI-CS managed in a Singaporean academic cardiology and cardiothoracic surgery center, over a 3-year period between 1 January 2017 and 31 December 2019. Consecutive adult patients (age ≥ 21 years) treated with IABP were identified from the hospital electronic record database. Patients who had IABP implanted for indications other than AMI-CS (such as IABP-supported percutaneous coronary intervention (PCI), IABP prior to open heart surgery and IABP use for mechanical complications following AMI) were excluded. Patients who had concurrent use of other MCS devices such veno-arterial (VA) ECMO were also excluded.

AMI was diagnosed according to the Fourth Universal Definition for Myocardial Infarction [16]. CS was defined by systolic blood pressure <90 mmHg for >30 min, or the need for inotropes or vasopressors to maintain a systolic pressure of >90 mmHg. Since our cohort of patients were treated by the managing physician with an MCS device, they would be classified as SCAI shock stage C and above [15]. The choice of inotropes or vasopressors was at the discretion of the managing physician. Patients who had evidence of ST-segment elevation on their electrocardiograms underwent immediate coronary angiogram and PCI; those without ST-segment elevation underwent urgent revascularization (within 24 h of admission). Patients were loaded pre-procedurally with aspirin 300 mg and ticagrelor 180 mg. They were subsequently recommended to continue aspirin 100 mg daily indefinitely and ticagrelor 90 mg twice a day for at least 12 months. All AMI-CS patients in our center invariably received IABP as an adjunctive therapy.

Demographic, clinical, laboratory, procedural, echocardiographic and outcome data were obtained from the hospital electronic records.

This study was approved by the Institutional Review Board, National Healthcare Group Domain Specific Review Board (reference number 2020/00265).

### 2.2. Study Outcomes

Primary outcome assessed was 30-day mortality. Demographic, clinical, laboratory, procedural and echocardiographic indices were evaluated. Safety outcomes evaluated included complications of IABP use, such as bleeding requiring transfusion, limb ischemia, IABP insertion site infection, and the need to remove IABP prematurely due to any device-related complication.

We also applied to this cohort our newly adopted institutional criteria for selection of patients with AMI-CS for advanced MCS using cVAD or VA-ECMO, looking at the proportion of patients who could have qualified for a higher degree of MCS (Supplementary Material).

### 2.3. Statistics

Comparison was made between patients who survived beyond 30 days and those who did not. Data was presented as mean ± standard deviation or frequency (%) and exploratory analyses were performed with the Mann–Whitney test and the chi-squared test. A backward elimination procedure (removal *p* > 0.05) was implemented to identify the final logistic regression model(s) concerning 30-day mortality, based on the significant predictors identified in the exploratory analyses and with interpretability also incorporated as a criterion for model selection. A Hosmer–Lemeshow test was performed to ascertain the goodness of fit of the model(s). Analyzed with Stata MP V16, all statistical tests were conducted at 5% level of significance.

## 3. Results

**Figure 1:**
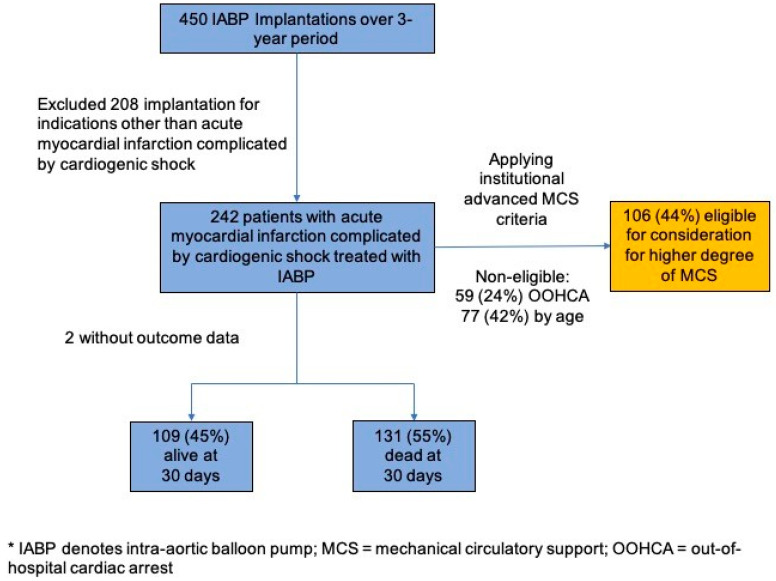
Patient flow and percentage of patients eligible for consideration for higher degree of mechanical circulatory support. (IABP denotes intra-aortic balloon pump; MCS = mechanical circulatory support; OOHCA = out-of-hospital cardiac arrest).

Over the 3-year period, 450 IABP implantation procedures were performed in our center, of which 208 procedures were excluded as implantations were for indications other than AMI-CS, including refractory angina, acute myocarditis, IABP supported PCI procedures, pre-CABG IABP support or repeat implantation procedure in the same hospital admission, and 242 unique patient-procedures were identified as having IABP implanted for AMI-CS and were included in the analysis. All patients had IABP implanted within 24-h of diagnosis of AMI-CS. Outcome data were not available for 2 patients, as they were transferred back to their home country to continue the management (Figure 1).

Baseline characteristics are presented in Table 1. Mean age of the cohort was 64.1 ± 12.4 years and the majority were males. Cardiovascular risk factors were prevalent, with hypertension seen in 147 (60.7%) and diabetes mellitus in 116 (47.9%). Patients were very ill on admission—110 (45%) suffered pre-IABP implantation cardiac arrest and 192 (83%) required inotropic or vasopressor support, prior to IABP implantation. More than half were attributed to STEMI, of which anterior STEMI was the most common. Revascularization was performed in 197 (81%) patients, mostly via PCI. More than half of the cohort did not survive beyond 30 days.

Table 2 compares characteristic of survivors versus non-survivors. Non-survivors were more likely to be non-smokers (69.5% versus 49.5%, *p* = 0.001), requiring inotrope or vasopressor prior to IABP implantation (87.5% versus 72.5%, *p* < 0.004) and to have undergone cardiac arrest prior to IABP implantation (61.6% versus 26.6%, *p* < 0.001), compared to survivors. Non-survivors had higher creatinine on admission (200.8 ± 173.6 versus 144.1 ± 153.1, *p* < 0.001), lower hemoglobin on admission (13.1 ± 2.2 versus 13.8 ± 2.3, *p* = 0.018), higher initial lactate levels (8.0 ± 5.3 versus 4.9 ± 4.3, *p* < 0.001), lower initial pH (7.12 ± 0.41 versus 7.30 ± 0.13, *p* < 0.001) and lower left ventricular ejection fraction (31.4 ± 13.3 versus 38.1 ± 11.9, *p* < 0.001). They had shorter duration of stay in the intensive care unit and hospital as compared to survivors (5.3 ± 5.9 versus 9.6 ± 9.0, and 6.3 ± 8.1 versus 20.6 ± 19.7, respectively, *p* < 0.001 for both).

Looking at predictors of 30-day mortality (Table 3), cardiac arrest prior to IABP implantation (adjusted odds ratio (aOR) 4.00, *p* < 0.001) and requirement of inotropes or vasopressors (aOR 2.42, *p* = 0.016) were found to be jointly significant in explaining 30-day mortality in this cohort of AMI-CS patients treated with IABP, while adjusting for STEMI (aOR1.35, *p* = 0.319). The significance of higher initial lactate reading (aOR 0.02, *p* = 0.04), lower pH on initial arterial blood gas (aOR 1.09, *p* = 0.040) and lower initial recorded hemoglobin level (aOR 0.83, *p* = 0.025) were also identified in the process, but their joint results were consolidated in a separate logit model in an attempt to isolate the influence of the predictors in the earlier analysis. The models were found to be satisfactory (Hosmer–Lemeshow test *p* > 0.05).

Complications related to IABP use were rarely encountered. They included four patients who developed bleeding requiring blood transfusion and three with limb ischemia. No patients developed IABP inserted site infection. In total, complications resulting in premature explant of IABP occurred in only 8 patients.

Finally applying our institutional advanced MCS eligibility criteria, 59 patients (24%) would have been disqualified due to out-of-hospital cardiac arrest at presentation. Of the remaining 183 patients, 77 (42%) were above the age of 65 years and would have been excluded. By current institutional workflow, 106 (44%) patients from this cohort would have qualified for evaluation for a higher degree of MCS, either with a cVAD or VA-ECMO (Figure 1).

## 4. Discussion

Our contemporary, real-world cohort of Asian AMI-CS patients, who were treated with IABP and early revascularization, had a 30-day mortality rate of 55%. Significant correlates of mortality included cardiac arrest and the need for inotrope or vasopressor prior to IABP implantation, higher lactate levels, lower pH and hemoglobin levels.

Our cohort of AMI-CS patients were sicker compared to those reported earlier; co-morbidities were more prevalent, a greater proportion suffered cardiac arrest or required inotrope/vasopressors prior to insertion of IABP, and they had higher lactate levels and lower pH for their first arterial blood gases. It is unsurprising that these factors were significant correlates of mortality; our findings are consistent with what was reported by Minha et al. in a small series of AMI-CS patients treated with IABP [17]. It is conceivable that this group of patients presented to hospital later and were therefore further along the CS cascade or had a very large infarct size with severe hemodynamic compromise. Another possibility would be that of delayed recognition and delayed treatment by physicians, resulting in patients spiraling down the CS cascade. An estimate of the extent of myocardial damage via peak troponin levels would have been ideal, but the information was not available for a proportion of them; this is consistent with real-world practice, where managing physicians would not have the laboratory findings at time of presentation. Regardless, the clinical predictors are helpful for rapid risk stratification in the Emergency Department and should prompt urgent resuscitation and institution of hemodynamic support measures.

In the latest SCAI Shock Stage Classification Expert Consensus Update, cardiac arrest with concern for anoxic brain injury was included as a risk modifier for poor prognosis [16]. Our cohort had a high rate (46%) of cardiac arrest prior to IABP implantation, with 54% of these episodes occurring before hospital arrival. In our cohort, prior cardiac arrest was noted to be associated with poorer prognosis, consistent with the SCAI document suggestion. Targeted temperature management was previously routinely used in patients with out-of-hospital cardiac arrest in our center. Due to the retrospective nature of this registry, we do not have data on the proportion of subjects in our cohort who received this treatment. With a recent study having questioned the efficacy of targeted temperature management, further evaluation of this treatment modality should be carried out to assess its utility in addressing the risk modifier of cardiac arrest in the setting of AMI-CS [18].

Lower hemoglobin level at presentation was an independent predictor of mortality in our patients. Anemia has been shown to predict worse outcomes in acute myocardial infarction and chronic stable ischemic heart disease [19]. Not surprisingly, this association was also seen in our AMI-CS cohort.

An interesting finding was that non-survivors were more likely to be non-smokers. This is counter-intuitive, given the strong correlation between cigarette smoking and coronary heart disease. Ironically, this could be attributed to excellent public education on the risks of smoking. Understanding their own risks, smokers possibly sought medical help earlier upon recognition of symptoms of AMI. In contrast, non-smokers could have had the misconception that they were at lower risk of cardiovascular disease and hence dismissed their symptoms, delaying presentation to a healthcare facility. Another possible explanation is non-disclosure by smokers in the cohort. This finding is significant, as many countries in Asia have high prevalence of smoking among men [20]. As Asian countries embark on public health campaigns to reduce smoking prevalence, we must not neglect health education to non-smokers, who can be lulled into a false sense of security due to their non-smoking status.

Non-survivors were also found to have shorter intensive care unit and hospital stays. This was due to cases with very early mortality, where severely ill patients either died in the cardiac catheterization laboratory after IABP implantation, or soon after transfer to the intensive care unit.

Another previous study from Europe looking at predictors of mortality in a cohort of patients with AMI-CS treated with IABP and PCI identified age, vasopressor use, resuscitation before PCI, acute renal failure and IABP implantation after PCI as independent predictors of in-hospital mortality in AMI-CS patients [21]. Except for the last predictor, our study result was congruous with these findings.

Knowledge of the above risk factors and predictors of poor outcomes is of paramount importance to patient care. Much effort can be put in to educating the public about symptoms of AMI, encouraging them to seek help early, avoiding unnecessary delays to definitive cardiology care. With early recognition and early treatment, we can hopefully avoid patients-at-risk spiraling down the irreversible CS cascade. Looking at the demographics of this patients, a high prevalence of cardiovascular pre-existing cardiovascular risk factors was also noted. This highlights the importance of risk factor management and preventive cardiology from the public health perspective.

There has been much controversy with regards to role of MCS devices in the management of AMI-CS. IABP use in AMI-CS has been under question even since the publication of the IABP-SHOCK II trial [22]. In that cohort of AMI-CS patients who underwent early revascularization, addition of IABP did not improve the primary endpoint of 30-day mortality, compared to best supportive care in the critical care unit. A subsequent systemic review of IABP strategy in AMI-CS suggested the beneficial effect of IABP on specific hemodynamic parameters, such as cardiac index, but again, failed to improve survival [23]. In the 2014 American Heart Association/American College of Cardiology guidelines for the management of patients with non-ST elevation acute coronary syndrome, routine use of IABP was no longer recommended in patients with CS [24]. This recommendation was similarly echoed in the 2017 European Society of Cardiology guidelines for management of STEMI [9].

IABP is still widely used in Asia, due to its low cost, ready availability of the device and long clinical experience. However, various national registries have reported a similar lack of efficacy of the IABP for the treatment of AMI-CS in the Asian population [25,26]. In place of the IABP, other advanced MCS devices may have a role in improving outcomes for AMI-CS patients. VA-ECMO has a long history of use in AMI-CS. However, many centers in Asia lack the financial and manpower support needed for an expensive VA-ECMO program. Furthermore, the VA-ECMO circuit has been long recognized to be non-physiological, increasing left ventricular afterload. All these factors result in the under-utilization of VA-ECMO as the mainstay MCS device in the treatment of AMI-CS in many parts of Asia, especially in countries with developing economies.

As for the role of the VA-ECMO in AMI-CS, the ECLS-SHOCK trial provided us with valuable insights [27]. In this trial, patients diagnosed with AMI-CS and who were planned for early revascularization, were randomized to receive VA-ECMO plus usual medical therapy or usual medical therapy alone. In the primary outcome of 30-day all-cause mortality, there was no difference between VA-ECMO and control. The rates of complications, such as bleeding or limb ischemia, were significantly greater in the VA-ECMO group. Although the result was disappointing, the cohort studied was very sick, with 78% of subjects suffering from cardiac arrest requiring resuscitation prior to randomization and 90% requiring mechanical ventilation. Furthermore, there was a high rate of crossover, with 13% of control patients eventually receiving VA-ECMO, as well as low rates of ventricular unloading (6%) during VA-ECMO treatment in the intervention arm.

Beyond VA-ECMO, the cVAD has emerged as the most popular device in the setting of AMI-CS, with its usage rising steadily since 2007, especially in North America [14]. The Impella^®^ family of cVADs are percutaneously inserted, micro-axial blood pumps which actively decompress the left ventricle, delivering blood to the ascending aorta in a continuous-flow manner. An early study comparing the cVAD against the IABP in a small cohort of AMI-CS patients showed that cVAD significantly improved cardiac power index at 30 min, compared to IABP [28]. Perceived hemodynamic advantage of the cVAD over IABP, coupled with the relative ease of implantation at the same setting of the PCI procedure, have contributed to the popularity of the cVAD.

Currently, the Impella CP^®^ is the most widely used cVAD in the setting of AMI-CS, adding up to 3.5 L per minutes of cardiac output to the failing heart. Despite superior cardiac output augmentation of the Impella CP^®^, it failed to improve 30-day and 6-month mortality, compared to the IABP in the IMPRESS trial [29]. A recent retrospective database analysis of MCS use in patients undergoing PCI in the United States even suggested that the Impella^®^ was associated with worse clinical outcomes and increased cost compared to IABP [14]. Similarly, a separate retrospective European registry comparing a matched Impella^®^ cohort with the historical IABP-SHOCK II cohort similarly showed increased rates of complications with Impella^®^ use, with no improvement in 30-day all-cause mortality [30]. Encouraging signals, however, have emerged from prospective registries looking at cohorts of carefully selected patients. The Detroit Cardiogenic Shock Initiative, which later gave rise to the National Cardiogenic Shock Initiative, proposed a team-based, protocolized management strategy in managing AMI-CS, with the Impella CP^®^ the MCS device of choice, when necessary [12,13]. In these two cohorts, reported survival to discharge were 76% and 72% respectively, marked improvement from previous AMI-CS cohorts. Although not randomized studies, these encouraging survival rates suggests the importance of careful patient selection and protocol adherence in MCS use for AMI-CS patients.

The recently published DanGer Shock trial provided the most compelling evidence for cVAD use in AMI-CS to date [31]. In this study, AMI-CS patients were randomized to receive the cVAD plus standard care or to standard care alone. Despite higher complication rates, patients in the cVAD arm had significantly lower mortality rates at 180-days (45.8%) compared to standard care arm (58.5%). This mortality benefit was seen despite a higher rate of complications in the cVAD arm, namely bleeding, limb ischemia, need for renal replacement therapy and sepsis. This trial was the first time that an MCS device was shown to confer mortality benefit when used in carefully selected AMI-CS patients. This will pave the way for more research into this area, potentially allowing us to further refine our patient selection process.

Our center is one of the first in Southeast Asia to adopt a ‘cardiogenic shock team’ strategy for the management of AMI-CS. Adapting the National Cardiogenic Shock Initiative protocol, we aim to carefully identify patients with AMI-CS who may benefit from early MCS device implantation, with the cVAD being the key tool. Using our current institutional selection criteria, 106 patients (44%) would have been eligible for evaluation for higher level of MCS. They would have undergone invasive hemodynamic assessment prior to primary PCI, for consideration of cVAD implantation or escalation of therapy to VA-ECMO. By adhering closely to published protocols and carefully selecting patients for these advanced MCS therapies, we hope to improve the survival of our population of Asian patients presenting with the dreaded AMI complication of CS.

With increasing interest and expertise in MCS in various Asian countries, coupled with more positive evidence in the field, we expect cVAD use to increase in Asia in the near future. We hope our experience with cVAD use in AMI-CS can provide a platform, for various healthcare systems across Asia, to formulate their own strategies in patient selection for cVAD implantation. This is after taking into account the high cost of the cVAD and the anticipated higher complication rates. For centres hoping to embark on a cVAD program, we propose that a multi-disciplinary ‘cardiogenic shock team’ should first be set up. This team should adopt currently available protocols, such as the NCSI protocol, to carefully evaluate every patient diagnosed with AMI-CS, for suitability for MCS device use [13]. Regardless of the MCS devices available to the cardiogenic shock team, this team-based, protocolized approach to management of AMI-CS can potentially already make a difference to patient outcomes.

As for the limitations of this study, this is a single-center study looking at a retrospective cohort of AMI-CS patients over 3 years. It is thus subject to the same limitations as any retrospective, observational study. Blood tests and echocardiograms were carried out at different time points, as per real-world practice, thus the timing of these investigations is a major confounder. Cardiac performance indices, such as measurements of cardiac index with the use of a pulmonary artery catheter, would also have been useful to better define cardiogenic shock in this cohort, but unfortunately these were not available as this was not routinely measured in this group of patients at that time. Although our cohort had IABP implanted within 24-h of AMI-CS diagnosis, the precise timing of IABP implantation, whether before or after PCI, was not available. We were also not able to retrospectively accurately classify out cohort of patients into the correct SCAI stages. Lastly, the percentage of AMI-CS patients who would have qualified for consideration of cVAD support instead, in our institution, is speculative and would warrant a formal registry study in the future.

In conclusion, our multi-ethnic Asian cohort showed that mortality for AMI-CS remains high despite early revascularization and IABP use. Prompt and protocolized treatment for AMI-CS patients, with possible early escalation of MCS therapy, has the potential to improve survival in this group of patients. In addition, public education on recognizing early signs and symptoms of AMI is equally important to avoid delay in timely definitive care, with the hope of stemming the progression to irreversible CS.

## Figures and Tables

**Table 1:** Cohort characteristics.

		All (n = 242)
**Patient demographics**		
Age (years)		64.1 ± 12.4
Gender—male (%)		213 (88.0%)
Height (m)		164.0 ± 8.0
Weight (kg)		68.0 ± 13.3
BMI (kg/m^2^)		24.7 ± 4.4
Smoking Status (%)		
	Non-Smoker	147 (60.7%)
	Ex-smoker	26 (10.7%)
	Current smoker	69 (28.5%)
Hypertension (%)		147 (60.7%)
Hyperlipidemia (%)		147 (60.7%)
Diabetes mellitus (%)		116 (47.9%)
End-stage renal disease on dialysis (%)		16 (6.6%)
Chronic obstructive lung disease (%)		8 (3.3%)
Obstructive sleep apnea (%)		3 (1.2%)
Prior ischemic heart disease (%)		77 (31.8%)
Prior PCI (%)		30 (12.4%)
Prior CABG (%)		12 (5.0%)
**Admission characteristics**		
STEMI (%)		
	STEMI	158 (65.3%)
	Non-STEMI	84 (34.7%)
Territory of ST elevation		
	Anterior	87 (55.1%)
	Non-anterior	71 (44.9%)
Cardiac arrest prior to IABP (%)		110 (45.5%)
	Out of hospital cardiac arrest (%)	59 (53.6%)
	In-hospital cardiac arrest (%)	51 (46.4%)
Inotrope/vasopressor use prior to IABP (%)		192 (80.3%)
Culprit coronary artery		
	Left anterior descending (%)	103 (42.6%)
	Right coronary artery (%)	46 (19.0%)
	Circumflex (%)	29 (12.0%)
	Others (%)	36 (14.9%)
	No clear culprit lesion identified (%)	28 (11.6%)
Systolic blood pressure before IABP (mmHg)		101.0 ± 32.2
Diastolic blood pressure before IABP (mmHg)		61.3 ± 18.6
Heart rate before IABP (bpm)		95.4 ± 25.2
PCI performed (%)		179 (74.0%)
CABG performed (%)		18 (7.4%)
Hemoglobin on admission (g/dL)		13.4 ± 2.2
Serum sodium (mmol/dL)		137.2 ± 4.7
Serum potassium (mmol/dL)		4.1 ± 0.7
Serum creatinine (mmol/dL)		173.0 ± 165.5
First lactate (mmol/dL)		6.58 ± 5.12
First pH on arterial blood gas		7.20 ± 0.33
**Echocardiographic characteristics**		
Left ventricular ejection fraction (%)		35.8 ± 12.3
Left ventricle end-diastolic dimension (mm)		49.0 ± 8.0
Left ventricle end-systolic dimension (mm)		38.2 ± 9.1
Septal wall thickness (mm)		10.4 ± 2.6
Posterior wall thickness (mm)		9.8 ± 2.1
Left ventricular mass index (g/m^2^)		103.8 ± 31.4
Right atrium area (cm^2^)		12.4 ± 3.2
Right ventricular basal dimension (mm)		22.7 ± 13.9
Right ventricular mid-ventricular dimension (mm)		17.2 ± 1.1
Right ventricular long axis dimension (mm)		43.3 ± 27.0
Mitral inflow E-wave deceleration time (ms)		146.5 ± 45.3
Septal E/E’		17.5 ± 8.5
**Outcomes**		
Duration of IABP support (days)		3.2 ± 2.3
Complications necessitating IABP removal (%)		8 (3.3%)
Bleeding requiring transfusion (%)		4 (1.7%)
Limb ischemia (%)		3 (1.2%)
IABP site infection (%)		0 (0.0%)
Need for inotropes/vasopressors (%)		212 (88.0%)
Need for mechanical ventilation (%)		198 (79.8%)
Duration of mechanical ventilation (days)		4.1 ± 5.1
Need for renal dialysis (%)		64 (26.5%)
Duration of ICU stay (days)		7.3 ± 7.8
Duration of hospitalization (days)		12.9 ± 16.1
Survival to 30-days (%)		109 (45.0%)

(BMI denotes body surface area; CABG = coronary artery bypass graft; IABP = intra-aortic balloon pump; ICU = intensive care unit; PCI = percutaneous coronary intervention; STEMI = ST-elevation myocardial infarction).

**Table 2:** Characteristics of 30-day survivors versus non-survivors (n = 240).

	Survivors (n = 109)	Non-Survivors (n = 131)	*p*-Value
**Patient demographics**			
Age (years)	62.6 ± 12.4	65.2 ± 12.2	0.055
Gender—male (%)	99 (90.8%)	112 (85.5%)	0.207
Height (m)	164.7 ± 7.7	163.3 ± 8.4	0.207
Weight (kg)	67.2 ± 12.7	66.3 ± 14.2	0.457
BMI (kg/m^2^)	24.8 ± 4.5	24.7 ± 4.3	0.904
Smoking Status			0.001
Non-smoker	54 (49.5%)	91 (69.5%)	
Previous	11 (10.1%)	15 (11.5%)	
Current	44 (40.4%)	25 (19.1%)	
Hypertension (%)	60 (55.6%)	85 (64.9%)	0.142
Hyperlipidemia (%)	64 (58.7%)	81 (61.8%)	0.623
Diabetes mellitus (%)	50 (45.9%)	64 (48.9%)	0.645
End-stage renal disease on dialysis (%)	4 (3.7%)	12 (9.2%)	0.090
Chronic obstructive lung disease (%)	4 (3.7%)	4 (3.1%)	0.791
Obstructive sleep apnea (%)	1 (0.9%)	2 (1.5%)	0.672
Prior ischemic heart disease (%)	35 (32.1%)	41 (31.3%)	0.893
Prior PCI (%)	18 (16.5%)	12 (9.2%)	0.086
Prior CABG (%)	3 (2.8%)	9 (6.9%)	0.145
**Admission characteristics**			
STEMI (%)	70 (64.2%)	86 (65.6%)	0.488
Non-STEMI	39 (35.8%)	45 (34.4%)	
Anterior territory ST elevation? (%)			0.398
Anterior	34 (31.2%)	51 (38.9%)	
Non-anterior	36 (33.0%)	35 (26.7%)	
Cardiac arrest prior to IABP (%)	29 (26.6%)	80 (61.1%)	<0.001
Out of hospital cardiac arrest (%)	16 (53.3%)	42 (51.9%)	0.890
In-hospital cardiac arrest (%)	13 (11.9%)	38 (29.0%)	0.003
Inotrope/vasopressor use prior to IABP (%)	79 (72.5%)	112 (87.5%)	0.004
Culprit coronary lesion			0.488
No	11 (10.1%)	17 (13.0%)	
Yes	98 (89.9%)	114 (87.0%)	
Systolic blood pressure before IABP (mmHg)	103.6 ± 33.7	98.3 ± 30.9	0.220
Diastolic blood pressure before IABP (mmHg)	63.0 ± 18.5	59.5 ± 18.5	0.230
Heart rate before IABP (bpm)	92.0 ± 23.5	98.1 ± 26.4	0.067
PCI performed (%)	85 (78.0%)	93 (71.0%)	0.218
CABG performed (%)	14 (12.8%)	4 (3.1%)	0.004
Hemoglobin on admission (g/dL)	13.76 ± 2.25	13.12 ± 2.18	0.018
Serum sodium (mmol/ dL)	137.18 ± 3.91	137.30 ± 5.37	0.709
Serum potassium (mmol/dL)	4.01 ± 0.67	4.23 ± 0.79	0.048
Serum creatinine (mmol/dL)	144.1 ± 153.1	200.8 ± 173.6	<0.001
First lactate (mmol/dL)	4.85 ± 4.30	8.01 ± 5.31	<0.001
First pH on arterial blood gas	7.30 ± 0.13	7.12 ± 0.413	<0.001
**Echocardiographic characteristics**			
Left ventricular ejection fraction (%)	38.1 ± 11.9	31.4 ± 12.3	<0.001
Left ventricle end-diastolic dimension (mm)	49.08 ± 7.41	48.80 ± 9.12	0.633
Left ventricle end-systolic dimension (mm)	38.1 ± 8.5	38.5 ± 10.3	0.930
Septal wall thickness (mm)	10.5 ± 2.6	10.1 ± 2.5	0.378
Posterior wall thickness (mm)	9.9 ± 2.3	9.5 ± 1.8	0.105
Left ventricular mass index (g/m^2^)	106.2 ± 31.7	98.8 ± 29.7	0.169
Right atrium area (cm^2^)	12.6 ± 3.1	12.0 ± 3.4	0.080
Right ventricular basal dimension (mm)	22.1 ± 14.0	23.7 ± 14.0	0.521
Right ventricular mid-ventricular dimension (mm)	16.5 ± 10.7	18.6 ± 11.8	0.317
Right ventricular long axis dimension (mm)	40.9 ± 26.2	47.1 ± 28.4	0.169
Deceleration time (ms)	146.9 ± 45.3	145.7 ± 46.0	0.859
Septal E/E’	16.7 ± 7.6	19.0 ± 10.1	0.284
**Outcomes**			
Duration of IABP support (days)	3.5 ± 2.3	2.9 ± 2.2	0.003
Complications necessitating IABP removal (%)	3 (2.8%)	5 (3.8%)	0.647
Bleeding requiring transfusion (%)	3 (2.8%)	1 (0.8%)	0.231
Limb ischemia (%)	1 (0.9%)	2 (1.5%)	0.672
IABP site infection (%)	0 (0.0%)	0 (0.0%)	NA
Need for inotropes/ vasopressors (%)	86 (79.6%)	125 (95.4%)	<0.001
Need for mechanical ventilation (%)	68 (62.4%)	124 (94.7%)	<0.001
Duration of mechanical ventilation (days)	4.3 ± 5.7	4.0 ± 4.6	0.424
Need for renal dialysis (%)	21 (19.3%)	43 (32.8%)	0.018
Duration of ICU stay (days)	9.6 ± 9.0	5.3 ± 5.9	<0.001
Duration of hospitalization (days)	20.6 ± 19.7	6.3 ± 8.1	<0.001

(BMI denotes body surface area; CABG = coronary artery bypass graft; IABP = intra-aortic balloon pump; ICU = intensive care unit; PCI = percutaneous coronary intervention; STEMI = ST-elevation myocardial infarction).

**Table 3:** Multiple logistic regression analysis of mortality at 30-days.

(a)			
Predictor	Adjusted Odds Ratio	95% C.I.	*p*-Value
Cardiac arrest prior to IABP	4.002	2.279–7.028	<0.001
Inotrope/vasopressor use prior to IABP	2.419	1.180–4.962	0.016
STEMI	1.347	0.750–2.420	0.002
**(b)**			
**Predictor**	**Adjusted Odds Ratio**	**95% C.I.**	***p*-value**
First pH on arterial blood gas	0.024	0.002–0.314	0.004
First lactate (mmol/dL)	2.419	1.004–1.192	0.040
Hemoglobin level on admission (g/dL)	0.833	0.711–0.978	0.025

(CI denotes confidence interval; IABP = intra-aortic balloon pump; STEMI = ST elevation myocardial infarction).

## Data Availability

The data presented in this study are available on request from the corresponding author due to patient confidentiality.

## Supplementary Materials

The following supporting information can be downloaded at: https://www.mdpi.com/article/10.3390/life14050577/s1.

## Abbreviations List

AMI	acute myocardial infarction
CABG	coronary artery bypass graft
CS	cardiogenic shock
cVAD	catheter-based ventricular assist device
ECMO	extracorporeal membrane oxygenation
IABP	intra-aortic balloon pump
MCS	mechanical circulatory support
OOHCA	out-of-hospital cardiac arrest
PCI	percutaneous coronary intervention
SCAI	Society for Cardiovascular Angiography and Intervention
STEMI	ST-elevation myocardial infarction
