# Supplementary material for: Predictors of Mortality in Acute Myocardial Infarction Complicated by Cardiogenic Shock despite Intra-Aortic Balloon Pump: Opportunities for Advanced Mechanical Circulatory Support in Asia
Source: Life (Basel). 2024 Apr 30;14(5):577. doi: 10.3390/life14050577 (PMC11122050; doi:10.3390/life14050577)
Supplement: Supplementary file 1 [file life-14-00577-s001.zip › life-2824961-supplementary.pdf]

Supplemental Material

Institutional Mechanical Circulatory Support Protocol (Excerpt)

Aims
- Rapid identification of patients with acute myocardial infarction complicated by cardiogenic shock
- Provide a guide for early initiation of appropriate mechanical circulatory support (MCS) for eligible patients
- Standarisation of patient selection for MCS device implantation prior to revascularisation
- Standarisation of patient care in the coronary care unit (CCU) after implantation of the Impella catheter-based ventricular assist device (cVAD)
- Decision-making guide for weaning of Impella cVAD ('Bridge to recovery')

Indications

Age <65 years **AND one of following clinical scenarios**
1. Acute myocardial infarction complicated by cardiogenic shock
    - Definition
        - Diagnosis of acute myocardial infarction (AMI)
            - Ischaemic symptoms of AMI
            - ECG and/or biomarker evidence of AMI (STEMI/ NSTEMI)
        - **AND** cardiogenic shock
            - Systolic blood pressure (SBP) ≤90 mmHg at baseline or requiring inotropes/ vasopressors to maintain SBP ⩾90 mmHg and
            - Evidence of end-organ hypoperfusion (cool extremities, oliguria, lactic acidosis)
2. Performance of high-risk complex PCI
    - Criteria include, but not limited to
        - Severe left ventricular (LV) systolic dysfunction with possible coronary flow interruption in a major branch
        - High risk of no-reflow (eg. vein graft intervention, rotablation, orbital atherectomy)
        - Chronic total occlusion (CTO) with retrograde approach
        - Last remaining major vessel
3. Myocarditis with anticipated recovery or bridge to definitive management

Contra-indications
- Evidence of anoxic brain injury
- Unwitnessed out of hospital cardiac arrest or any cardiac arrest in which return of spontaneous circulation (ROSC) is not achieved in 30 mins
- Septic, anaphylactic, hemorrhagic, and neurologic causes of shock
- Other causes of shock/hypotension outside the eligible clinical scenarios (pulmonary embolism, pneumothorax, pericardial tamponade)
- Active bleeding
- Mechanical complications of AMI (ventricular septal defect, acute papillary muscle rupture)
- Known LV thrombus
- Mechanical aortic prosthetic valve
- Presence of severe aortic stenosis
- Presence of moderate to severe aortic regurgitation

- Known history of severe peripheral vascular disease
- Contraindication to intravenous systemic anticoagulation

Right Heart Catheterisation

- Complete right heart catheterization to be performed, to obtain following parameters
    - Right atrial pressure
    - Right ventricular (RV) pressure
    - Pulmonary artery pressure
    - Pulmonary capillary wedge pressure (PCWP)
        - Left ventricular end-diastolic pressure (LVEDP) if unable to obtain PCWP
    - Mixed venous oxygen saturation (from pulmonary artery)
    - Cardiac output/ cardiac index (using Fick method)
- Calculate cardiac power output and pulmonary artery pulsatility index
    - Cardiac Power Output (CPO)
        - [Mean Arterial Pressure (MAP) x Cardiac Output (CO)] / 451
    - Pulmonary Artery Pulsatility Index (PAPi)
        - [Pulmonary Artery Systolic Pressure (PASP) – Pulmonary Artery Diastolic Pressure (PADP)] / [Right Atrial Pressure (RAP)]
- Leave pulmonary artery catheter in-place

Selection of Mechanical Circulatory Support

- Place Impella® device for isolated LV failure and cardiogenic shock prior to performing primary PCI
    - Cardiac index <2.2 L/min/m^2 **AND**
    - PCWP (LVEDP if unable to obtain PCWP) >15 mmHg **AND**
    - PAPi >0.9
- Consider veno-arterial extra-corporeal membrane oxygenation (VA-ECMO) if bi-ventricular failure
    - Cardiac index <2.2 L/min/m^2 **AND**
    - PCWP (LVEDP if unable to obtain PCWP) >15 mmHg **AND**
    - PAPi <0.9
- IABP may be considered as adjunct support if selection criteria for Impella® and VA-ECMO are unmet

Coronary Angiogram and Coronary Intervention

- Perform coronary angiogram and intervene on culprit lesion
    - On maximum Impella® support (P8 support)
- Interventional cardiologist to decide on the intervention of other incidental lesions

Emergency CABG

- If coronary lesions are not amenable to PCI and patient has on-going myocardial ischemia
    - On maximum Impella® support (P8 support)
- Discussion with cardiothoracic surgeon on Impella support intra-operatively and post-CABG management
